# Macronutrient Modulation in Metabolic Dysfunction-Associated Steatotic Liver Disease—the Molecular Role of Fatty Acids compared with Sugars in Human Metabolism and Disease Progression

**DOI:** 10.1016/j.advnut.2025.100375

**Published:** 2025-01-20

**Authors:** Sinéad M Mullin, Aidan J Kelly, Méabh B Ní Chathail, Suzanne Norris, Christopher E Shannon, Helen M Roche

**Affiliations:** 1 School of Public Health, Physiotherapy and Sport Science, and Institute of Food and Health, University College Dublin, Belfield, Dublin, Ireland; 2 Nutrigenomics Research Group, UCD Conway Institute, University College Dublin, Belfield, Dublin, Ireland; 3 School of Medicine, Trinity College Dublin, Dublin, Ireland; 4 School of Medicine, University College Dublin, Belfield, Dublin, Ireland; 5 Institute for Global Food Security, Queen’s University Belfast, Northern Ireland

**Keywords:** MASLD, MASH, inflammation, fibrosis, fatty acids, carbohydrates, sugars, fiber, dietary intervention, precision nutrition

## Abstract

Metabolic dysfunction-associated steatotic liver disease (MASLD) is a significant public health concern, with its progression to metabolic dysfunction-associated steatohepatitis (MASH) and fibrosis leading to severe outcomes including cirrhosis, hepatocellular carcinoma, and liver failure. Whereas obesity and excess energy intake are well-established contributors to the development and progression of MASLD, the distinct role of specific macronutrients is less clear. This review examines the mechanistic pathways through which dietary fatty acids and sugars contribute to the development of hepatic inflammation and fibrosis, offering a nuanced understanding of their respective roles in MASLD progression. In terms of addressing potential therapeutic options, human intervention studies that investigate whether modifying the intake of dietary fats and carbohydrates affects MASLD progression are reviewed. By integrating this evidence, this review seeks to bridge the gap in the understanding between the mechanisms of macronutrient-driven MASLD progression and the effect of altering the intake of these nutrients in the clinical setting and presents a foundation for future research into targeted dietary strategies for the treatment of the disease.

**Statement of significance**

This review offers a novel perspective on the role of dietary fatty acids and fructose in driving the progression of MASLD to MASH and fibrosis by integrating the mechanistic insights obtained from preclinical work with evidence from human intervention studies.

## Introduction

Metabolic dysfunction-associated steatotic liver disease (MASLD), formerly known as nonalcoholic fatty liver disease (NAFLD) [1], is the hepatic manifestation of a constellation of dysfunctional metabolic features that includes insulin resistance, visceral adiposity, hyperlipidemia, reduced high-density lipoprotein cholesterol, and chronic inflammation [2]. MASLD is a heterogeneous disorder that is defined as the presence of hepatic steatosis in conjunction with one cardiometabolic risk factor, with the exclusion of known secondary causes [1]. MASLD is the most common chronic liver disease in the world; its global prevalence was estimated to be 30% in 2023, with significant regional variation [3]. Rates of MASLD have increased in parallel with those of obesity, and as a continued increase in the global prevalence of obesity is anticipated, the burden of MASLD is set to become enormous. Indeed, MASLD is the fastest growing indication for liver transplantation in Western countries [4].

MASLD is a spectrum of diseases ranging from simple steatosis to metabolic dysfunction-associated steatohepatitis (MASH) and cirrhosis. MASH is characterized by steatosis with additional hepatocyte ballooning (a histological sign of hepatocyte injury) and lobular inflammation [5]. Although both simple MASLD and MASH can progress to fibrosis and eventually cirrhosis, the rate of progression is greater in MASH [6]. In addition, the incidence of hepatocellular carcinoma is 12-fold higher in MASH patients compared with steatosis-only MASLD patients [7], and progression of MASH is associated with increased all-cause and liver mortality [8,9]. Thus, it is important that the scientific community develops a thorough understanding of the factors that drive the differentiation of steatohepatitis from simple steatosis. This review attempts to go beyond the impact of excess dietary energy intake and obesity, discussing the potential differential mechanistic roles of macronutrients, specifically dietary fatty acids, and sugars, in the progression of MASLD to MASH and fibrosis, then providing an overview of interventional studies in which the effect of changes to the intake of these dietary triggers on MASLD progression have been investigated.

## Fatty Acids and Sugars in the Progression of MASLD to MASH and Fibrosis

The establishment of hepatic steatosis occurs due to an imbalance in the processes governing hepatic lipid accumulation, namely fatty acid uptake and de novo lipogenesis (DNL) relative to β-oxidation and lipid export in VLDL, as extensively reviewed [10]. A recent systematic review and meta-analysis of observational studies in humans demonstrated that total caloric intake was higher in people with MASLD compared with healthy controls [11]. Also, consumption of SFA was not significantly different between these groups, but fructose consumption was higher in people with MASLD. The synergistic effect of SFA and fructose on steatosis development is evidenced by the finding that in mice fed with fructose in addition to a high-fat Western diet, liver steatosis, and fibrosis were worse than in the absence of fructose, despite the diets being isocaloric [12]. Additionally, a similar experiment showed that the addition of fructose to a high-fat diet likely increases hepatic steatosis primarily by inhibiting β-oxidation, thus demonstrating a mechanistic basis for the recognized synergistic effect [13]. Nevertheless, our precise understanding of the impact of these individual dietary triggers in driving MASLD beyond steatosis, including whether deleterious synergistic effects are at play, remains limited.

The degree of hepatic steatosis is not directly correlated with the risk of progression to MASH and fibrosis. Rather, this progression requires other stressors or “hits” beyond steatosis that lead to the activation of liver-resident and circulating immune cells and hepatic stellate cells (HSC) [14]. These hits include endoplasmic reticulum (ER) stress, lipoapoptosis, and the release of extracellular vesicles (EV); increased intestinal permeability and changes to the gut microbiota; metabolic reprogramming; and oxidative stress and inflammasome activation. Somewhat unsurprisingly, given the close association of MASLD with general metabolic dysfunction and chronic inflammation, these processes also coexist with insulin resistance [15]. The mechanisms by which the dietary triggers implicated in MASLD progression—SFA and fructose—contribute to these processes are discussed in this section.

### The role of immune cells and HSC in MASLD progression

The population of the resident phagocytic cells of the liver, Kupffer cells, is expanded early in the progression of MASLD and before the infiltration of other immune cells [16]. These other circulating immune cells infiltrate the liver in response to proinflammatory cytokines, and the extent of their infiltration positively correlates with the severity of steatosis, hepatocyte ballooning, and fibrosis [17]. The infiltration of circulating bone marrow-derived monocytes (BMDM) is greatly upregulated upon liver injury and in response to high-fat feeding in mice, and these differentiate into pro-inflammatory M1 macrophages and promote neutrophil infiltration [[18], [19], [20]]. Furthermore, the number and phenotype of circulating immune cells are changed with MASLD progression, including an increase in the frequency of intermediate and nonclassical monocytes and changes to pattern recognition receptor (PRR) expression in cytotoxic T cells [21,22]. HSCs are normally quiescent in the healthy liver but are activated and differentiate into myofibroblasts, which produce extracellular matrix (ECM) proteins in response to molecular markers of liver damage and inflammation [23]. However, in MASLD, HSC activation and ECM production occur in excess of the rate of ECM degradation, resulting in overall accumulation [24]. There is also a secondary role for other ECM-producing cells in MASLD fibrosis, such as portal fibroblasts and vascular smooth muscle cells [24].

### ER stress, lipoapoptosis, and extracellular vesicles in MASLD progression

In hepatocyte cell lines, the accumulation of SFA due to the inhibition of its desaturation and its incorporation into triacylglycerol (TAG), normally cytoprotective processes, causes the saturation of the ER membrane [25,26]. This leads to the induction of ER stress responses, including the unfolded protein response, as well as the activation of intrinsic and extrinsic pathways of apoptosis [[25], [26], [27], [28]]. This process of ER saturation is attenuated by oleic acid, a MUFA [28], and the extrinsic pathway of apoptosis depends on the de novo synthesis of ceramide for the formation of lipid rafts [29], illustrating that the mix of lipid species in hepatocytes is important in influencing MASLD progression. Targeting hepatocyte lipid saturation, the starting point for this process is the strategy of the first approved therapeutic for noncirrhotic MASH with moderate to advanced liver fibrosis, resmetirom, a thyroid hormone receptor-β selective agonist [30].

Palmitate-induced hepatocyte ER stress and lipoapoptosis interact with immune cells via the release of EVs, including microvesicles, apoptotic bodies, and exosomes. These have been shown to stimulate the release of proinflammatory cytokines and death ligands from Kupffer cells and BMDMs, which induce hepatocyte apoptosis and pyroptosis, and, in the autophagy-deficient state that exists in MASLD, necrosis and further release of exosomes containing damage-associated molecular patterns (DAMPs) [20,[31], [32], [33]]. Therefore, the release of these EVs could set up a positive feedback loop by promoting the release of proinflammatory prodeath mediators from immune cells, further driving lipoapoptosis. EVs from lipid-stressed hepatocytes also promote the infiltration of immune cells. They induce the expression of adhesion molecules and IL-1β by endothelial cells and cause monocyte adhesion via integrin-β1 [[34], [35], [36]], promote angiogenesis via vanin-1 [37], and induce macrophage chemotaxis via chemokine (C-X-C motif) ligand 10 [38]. EV production requires the de novo synthesis of ceramide from palmitate [39]. Additionally, palmitate-derived lipid species promote infiltration of immune cells. Sphingosine-1-phosphate (S1P) induces macrophage chemotaxis, which is potentiated by palmitate-induced S1P receptor expression on macrophages [40]. Hepatocytes treated with cholesterol or oxidized LDL (oxLDL), which are raised in MASLD and in response to SFA feeding, caused polarization of macrophages to the M1 phenotype via EVs containing microRNA-122-5p [41].

Palmitate-induced EVs also contribute to the development of fibrosis in MASLD [42]. In addition to inducing the production of profibrogenic transforming growth factor β1 (TGF-β1) by macrophages, hepatocyte apoptotic bodies are also engulfed by HSCs, causing their activation [43]. This activation is mediated, in part, by microRNAs that suppress peroxisome proliferator-activated receptor gamma expression, inhibit mitophagy, and upregulate the AKT1 signaling pathway in HSCs [[44], [45], [46]]. Activated HSCs themselves also release EVs, which suppress antifibrogenic signals in neighboring HSCs [47]. This demonstrates the complexity of this cell-to-cell signaling method, which derives from the multitude of sources of EVs, their varied cargoes, and their changing composition and abundance throughout the progression of MASLD. HSCs also show greater ER stress in more severe fibrosis, which is critical for their activation. PKR-like endoplasmic reticulum kinase (PERK) activates SMAD2, a canonical downstream messenger of TGF-β1, following ER stress [48], and SMAD2 itself signals through inositol-requiring enzyme 1α (IRE1α) to cause HSC activation [49]. Furthermore, ER stress in hepatocytes causing activation of stimulator of interferon genes (STING) and subsequent apoptosis has been shown to be necessary for fibrosis in a mouse model of liver injury, demonstrating that liver fibrosis is not solely an inflammation-driven process [50]. STING activation also promotes the formation of p62-containing protein inclusions in hepatocytes under SFA lipotoxicity, which are known to drive fibrosis and carcinogenesis. Crucially, the formation of these inclusions was almost completely inhibited by the addition of oleic acid and docosahexaenoic acid, suggesting that the relative abundance of SFA and unsaturated fatty acids (UFA) in the diet may influence MASLD progression [51]. These pathways demonstrate potential mechanisms by which palmitate-induced ER stress promotes fibrosis in MASLD. Lastly, exosomes containing glucose transporter 1 (GLUT1) and pyruvate kinase isozymes M1/M2 from HSCs transmit metabolic reprogramming signals to other HSCs and nonparenchymal cells by promoting the glycolytic shift that is necessary to activate these cells [52,53].

Fructose consumption also promotes the development of inflammation and fibrosis in MASLD, by increasing ER stress. Fructose greatly increases the expression of enzymes in the DNL pathway via sterol response element-binding protein 1c, which may overload the ER and result in misfolding, with an additive effect on ER stress [54]. Furthermore, X-box binding protein 1 (XBP1), which is activated by IRE1α upon ER stress, is itself a transcription factor for several lipogenic enzymes, compounding the problem [55]. Increased XBP1 expression also increases macrophage and HSC activation by inhibiting BCL2/adenovirus E1B interacting protein 3-mediated mitophagy [56]. Moreover, IRE1α was shown to cleave microRNA-150, removing the suppression of α-smooth muscle actin in myofibroblasts [57]. The expression of ketohexokinase-C is also upregulated by uric acid derived from fructolysis, thereby further increasing the rate of fructolysis [58] and global protein acetylation, which causes protein misfolding and contributes to the development of ER stress, leading to proinflammatory signaling, infiltration by circulating immune cells, and fibrogenesis [59].

There is a possibility that hepatic steatosis resulting from hypercaloric diets containing SFA and fructose, rather than SFA and fructose directly, drives the progression of MASLD to MASH and fibrosis. However, the distinction between, on the one hand, the accumulation of SFA in TAG leading to hepatocyte steatosis and, on the other, the mechanistic role of SFA in initiating ER stress and its deleterious consequences is important, as it shows that SFA promotes MASLD progression independent of its role in the development of steatosis. This distinction also exists for fructose, as it is the induction of the translation of enzymes to facilitate DNL and the consequent overloading of the ER by these enzymes, not the steatosis that results from the induction of DNL, that triggers ER stress. Further research should focus on separating the contributions of SFA and fructose to steatosis and to MASLD progression.

### Gut microbiota and intestinal permeability in MASLD progression

In health, Kupffer cell responses to pathogen-associated molecular patterns (PAMPs), present in the liver and portal circulation due to the translocation of components of the gut microbiota, are controlled due to tight regulation of the toll-like receptor (TLR) signaling pathway [60]. Changes to the amount and composition of microbiota components in the portal circulation occur in MASLD due to intestinal barrier dysfunction and intestinal dysbiosis [61]. Studies of plasma and hepatic biopsies from patients with MASLD consistently show raised hepatic LPS concentrations in simple steatosis but report varying associations with MASLD progression [[62], [63], [64]]. Furthermore, there is no consensus on specific changes to the gut microbiome with MASLD progression [65], but the finding that the addition of *Lactobacillus paracasei* reduced Kupffer cell infiltration along with intestinal permeability in a MASH mouse model suggests that there is some causative role for gut-derived PAMPs in MASLD inflammation [66].

Chronic exposure to fructose may promote MASLD progression by increasing intestinal permeability, thereby allowing greater translocation of gut microbiota components into the portal circulation. In mice, 8-wk of high-fructose water consumption caused decreased duodenal expression of occludin, a component of enterocyte tight junctions, and induced TLR4-dependent MASH despite no change to duodenal microbiota [67,68]. Similarly, consumption of a “Western” diet for 3-mo by mice prone to ER stress increased colonic permeability, with associated changes in expression of tight junction proteins and subsequent steatohepatitis [69]. Mechanistically, fructose-induced intestinal permeability is caused by increased oxidative and nitrative stress, which lead to the ubiquitination and degradation of tight junction, adherens junction, and desmosome proteins [70], and by the inhibition of post-translational modification of proteins, leading to ER stress [69]. Consistent with TLR4 activation by LPS, these intestinal changes are associated with increased hepatic expression of chemokine (C-C motif) ligand 2 (CCL2) and of markers of M1 macrophages and with increased lipid peroxidation [71]. High-fat feeding can also increase intestinal permeability by reducing the expression of tight junctions, possibly by increasing bile acid-induced ER stress in intestinal stem cells [72,73]. HSCs also respond to LPS via TLR4 by increasing the production of TGF-β1 and col1A1; they also become more responsive to TGF-β1 due to downregulation of its pseudoreceptor bone morphogenetic protein and activin membrane-bound inhibitor [74]. This is worsened by free cholesterol loading of HSCs, which inhibits TLR4 endocytosis and thereby exacerbates HSC activation and responsiveness to TGF-β1 [75].

Fructose and fat both alter the microbiota composition. High-fat feeding in mice caused a decrease in the ratio between Bacteroides and Firmicutes and a large increase in Proteobacteria abundance, associated with greater hepatic inflammasome activation and fibrosis, potentially by causing greater production of LPS [76]. Similarly, in rats, fructose feeding increased the abundance of Coprococcus and Ruminococcus, the concentration of LPS in the portal circulation, and hepatic inflammation, all of which were attenuated by antibiotic treatment and fecal transplant from feed pellets-fed rats [77,78]. Conversely, the addition of fructose to high-fat feeding in mice had no effect on intestinal permeability and reduced dysbiosis, associated with altered Kupffer cell phenotype and increased hepatic lymphocyte recruitment [79]. These results suggest that dietary components may modulate the microbiota and intestinal permeability, though there is a need for further investigation into this complex field to elucidate the direct and indirect mechanisms at play. Indeed, it is probable that metabolites derived from the gut microbiome mediate their effects on intestinal permeability and inflammation; however, in terms of brevity, this is not addressed herein [80].

### Metabolic reprogramming in MASLD progression

Metabolic adaptations to allow changes to transcription and protein synthesis, known as metabolic reprogramming, are implicated in MASLD inflammation and fibrosis, which are contributed to by dietary triggers. Initially, SFAs were thought to induce proinflammatory signaling via TLR4 with a similar magnitude to that induced by LPS [81]. However, more recent modeling of palmitate binding to TLR4 showed that TLR4 dimerization, TLR4 endocytosis, and nuclear factor kappa-light-chain-enhancer of activated B cells activation, necessary steps in TLR4 signaling, did not occur in palmitate-treated BMDM [82]. Palmitate proinflammatory signaling was abolished by TLR4 knockout but restored with TLR2 or TLR3 priming; Lancaster et al. suggested that metabolic reprogramming induced by PAMP via TLR signaling, including a reduction in fatty acid oxidation, alters the metabolism of SFAs and leads to ER stress and c-Jun N-terminal kinase activation via plasma-bound Rho GTPases [82]. In contrast, UFAs have been shown to attenuate this proinflammatory effect, likely by reducing ER stress and by activating AMP-activated protein kinase, thereby inhibiting NLR family pyrin domain containing 3 (NLRP3) inflammasome activation [83,84]. Uptake of SFA by macrophage scavenger receptor 1 causes the release of tumor necrosis factor-α (TNFα) via c-Jun N-terminal kinase phosphorylation, although this is not the case for UFA [85]. The importance of lipid handling in macrophages in MASLD is further illustrated by the findings that inhibition of macrophage DNL in a high-fat diet inhibited LPS-induced cytokine production [86], and that pan-agonism of peroxisome proliferator-activated receptors, which regulate lipid metabolism, reduced palmitate-induced cytokine production by murine BMDMs and patient monocytes[87]. Finally, as with macrophages, the activation of HSCs in MASLD progression involves metabolic reprogramming, which is reviewed by Horn and Tacke [52] and is, therefore, sensitive to the dietary triggers implicated in MASLD progression.

Fructose potentiates the inflammatory response to PAMP via metabolic reprogramming. Fructose causes mammalian target of rapamycin complex 1 (mTORC1) activation via glutamine metabolism and dihydroxyacetone phosphate, a product of fructose metabolism by aldolase B, causing reduced glycolysis, oxidative phosphorylation, and LPS-induced cytokine translation and release [88]. Even in the absence of PAMP, acute exposure of human dendritic cells to fructose increased cytokine expression associated with the production of advanced glycation end-products (AGE) and the upregulation of receptors for advanced glycation end-products and glycolysis [89]. However, the production of uric acid and oxidative stress from fructolysis, which is discussed in the next section, maybe more significant fructose-related factors in MASLD progression than this metabolic reprogramming.

### Oxidative stress and inflammasome activation in MASLD progression

Oxidative stress and inflammasome activation due to the overabundance of dietary triggers in MASLD is another factor that promotes the progression of the disease. Reactive oxygen species (ROS) from lipid-induced ER stress and from cholesterol-induced mitochondrial stress sensitize hepatocytes to apoptosis [90,91]. ROS also promote innate immune signal transduction and thereby increase the production of proinflammatory and profibrogenic cytokines [92]. Consistent with this, palmitate was shown to induce the release of mitochondrial DNA, which activates NLRP3, from Kupffer cell mitochondria with electron transport chain (ETC) impairment [93]. Furthermore, expression of STING, which is activated by mitochondrial DNA, is increased in macrophages of MASLD patients and induces inflammatory signaling and HSC activation [94]. Dietary cholesterol also increases mitochondrial ROS production by perturbing the fluidity of the mitochondrial inner membrane [95] and inhibiting the transport of glutathione into mitochondria [96,97]. This effect of cholesterol may be compounded in the inflammatory milieu of obesity and MASLD; SFAs impair the ability of HDL to efflux cholesterol from macrophages, potentially due to the increased association of acute-phase inflammatory proteins with HDL particles, and cause hepatic cholesterol accumulation, compared with MUFA [98].

Serum uric acid increases with MASLD progression [99], and the accumulation of uric acid and other DAMPs paralleled the progression of MASLD in a high-fat, high-fructose, high-cholesterol mouse model associated with the hepatic upregulation of NLRP3, IL-1β, TNFα, and CCL2 [100]. The production of uric acid from fructolysis is itself a source of ROS [101], but uric acid also causes the translocation of nicotinamide adenine dinucleotide phosphate oxidase (NOX), a producer of ROS to mitochondria, resulting in inhibition of the tricarboxylic acid (TCA) cycle and the ETC, increased DNL, and mitochondrial DNA damage [102]. ROS produced by NOX mediate the induction of collagen by TGF-β1 and by apoptotic body engulfment in HSCs [103,104]). Also, ROS produced by uric acid-activated extra-mitochondrial NOX cause ER stress, leading to further DNL [105]. Therefore, DNL and uric acid-induced oxidative and ER stress may form a positive feedback loop in high-fructose settings, promoting MASLD progression. ROS from uric acid crystals activates NLRP3 via thioredoxin-interacting protein [106], and uric acid also activates NLRP3 in HSCs, upregulating TGF-β1 and collagen expression [107]. Uric acid also activates NLRP3 by inhibiting AMP-activated protein kinase-mediated autophagy [108], a mechanism shared by SFA [109]. NLRP3 activates HSCs and promotes immune cell infiltration by triggering pyroptosis of hepatocytes via gasdermin, causing a huge release of DAMP [110]. Indeed, NLRP3 was shown to be essential for fibrosis development in a mouse model of MASH and has higher expression in advanced MASLD in humans [111].

### Summary

**FIGURE 1:**
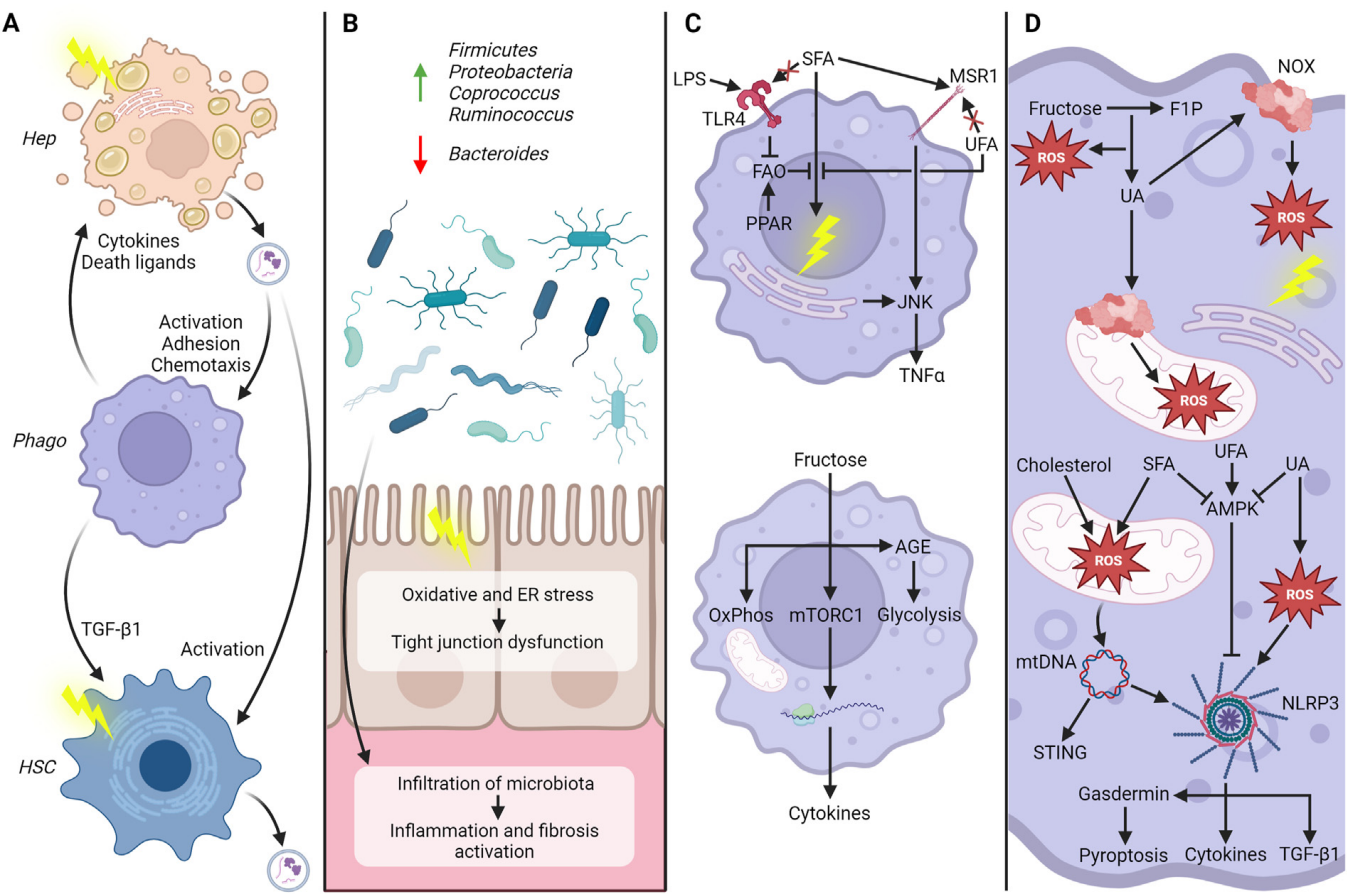
Dietary triggers in the progression of MASLD to MASH and fibrosis. (A) The saturation of the ER membrane by SFA and the excessive induction of DNL protein expression by fructose activate the unfolded protein response, leading to lipoapoptosis of hepatocytes. EVs released by dying hepatocytes activate resident and circulating phagocytes, which release cytokines and death ligands that perpetuate this process. ER stress, TGF-β1 from phagocytes, and EVs from hepatocytes activate HSCs, which themselves release EVs that transmit activating signals to other HSCs and nonparenchymal cells. (B) Diets high in fructose or SFA alter the gut microbiota composition and increase intestinal permeability by disrupting tight junction integrity in enterocytes under ER and oxidative stress, thereby allowing the infiltration of microbiota components into the portal circulation and the resulting activation of inflammation and fibrosis. (C) TLR4 signaling, previously thought to be activated by SFA, inhibits β-oxidation of SFA, causing ER stress; this is opposed by UFA and PPAR signaling. SFA are also bound by phagocyte scavenger receptors, causing the release of proinflammatory cytokines. Fructose increases OxPhos and glycolysis via the production of AGE and upregulates the translation of cytokines via mTORC1 activation. (D) Uric acid is produced by fructolysis and activates NOX, leading to the production of ROS and mitochondrial and ER stress. Uric acid also activates NLRP3 via ROS production and the inhibition of AMPK, a mechanism shared by SFA and opposed by UFA. In mitochondria, electron transport chain dysfunction, altered membrane fluidity, and impaired antioxidant mechanisms due to SFA and cholesterol lead to ROS production and the release of mtDNA, which activates STING and NLRP3. NLRP3 promotes the release of cytokines and TGF-β1 and the activation of gasdermin, which causes pyroptosis. AGE, advanced glycation end-product; AMPK, AMP-activated protein kinase; ER, endoplasmic reticulum; F1P, fructose-1-phosphate; FAO, fatty acid oxidation; Hep, hepatocyte; HSC, hepatic stellate cell; JNK, c-Jun N-terminal kinase; MSR1, macrophage scavenger receptor 1; mtDNA, mitochondrial DNA; mTORC1, mammalian target of rapamycin complex 1; NLRP3, NLR family pyrin domain containing 3; NOX, NADPH oxidase; OxPhos, oxidative phosphorylation; Phago, phagocyte; PPAR, peroxisome proliferator-activated receptor; STING, stimulator of interferon genes; ROS, reactive oxygen species; SFA, saturated fatty acid; TGF-β1, transforming growth factor β1; TLR4, toll-like receptor 4; TNFα, tumor necrosis factor α; UA, uric acid; UFA, unsaturated fatty acid. Created with BioRender.com

In summary, dietary triggers promote the progression of MASLD to MASH and fibrosis in 4 main ways (Figure 1). Firstly, the initiation of ER stress by SFA and fructose-induced DNL causes lipoapoptosis of hepatocytes and the release of EVs that activate phagocytes and HSCs, which in turn release cytokines, death ligands, and further EVs that perpetuate this process. Secondly, SFA and fructose alter the gut microbiota composition and increase intestinal permeability, allowing the infiltration of microbiota components into the portal circulation, where they promote the activation of inflammation and fibrosis. Thirdly, inflammatory signaling pathways interact with fatty acid metabolism to increase the production of proinflammatory cytokines, and fructose increases the translation of cytokines and promotes the metabolic reprogramming that is necessary to support cytokine production by phagocytes. Lastly, in hepatocytes, phagocytes, and HSCs, the excessive production of ROS due to an overabundance of fructose, SFA, and cholesterol, the resulting organelle dysfunction, and the activation of inflammasomes and other innate immune sensors that follow lead to pyroptosis, cytokine release, HSC activation, and fibrosis. Understanding the mechanisms underpinning the individual specific diet stressor compared with synergistic effects is important with a view to identifying potential therapies to interrupt such pathways.

## Modulating Dietary Intake for the Management of MASLD: Evidence from Human Dietary Intervention Studies

Whereas robust observational evidence for the specific deleterious roles of SFA and fructose in MASLD in humans are not yet consistently forthcoming [15], the wide-ranging insights into the contributions of SFA and fructose to the mechanisms that drive MASLD progression provide a strong footing to consider the potential efficacy of removing these dietary triggers to prevent the progression of MASLD to MASH and fibrosis. Diet and lifestyle changes are currently the first-line therapy for the management of hepatic steatosis. To this end, a number of dietary approaches have been trialed, and this area has been extensively reviewed [[112], [113], [114]]. The consensus is that in the context of a hypocaloric diet and where significant weight loss (7%–10%) is achieved, there is no superior approach. However, the field requires greater understanding of whether sensitivity to certain dietary stressors, such as SFA and fructose, and removal thereof, is an effective therapeutic approach. Indeed, the Mediterranean diet, with substitution of SFA for MUFA, is often highlighted for its additional cardiometabolic benefits, reducing cardiovascular disease risk, a frequent cause of mortality in patients with MASLD [112,113]. Within the context of Precision Nutrition, there is no doubt that there is variable efficacy to dietary interventions, wherein certain individuals with distinct metabolic phenotypes (pertaining to the metabolic syndrome (MetS)) respond to dietary interventions, typically 40%, but others do not [[115], [116], [117]]. It is probable that the same paradigm exists for MASLD, given its overlap with MetS.

Indeed, for the treatment of MASH, it is becoming increasingly recognized that a more targeted approach is required, not least because patients struggle to achieve and sustain the required weight loss of >10% body weight. Furthermore, growing evidence suggests that specific fatty acids and carbohydrates could play a significant role in both driving and attenuating inflammation and fibrosis associated with MASH, suggesting that dietary changes may have benefits beyond weight loss. However, how these mechanistic insights translate into practical dietary interventions remains unclear.

As such, this section reviews evidence from intervention studies in which dietary fatty acid and carbohydrate intake has been altered and the impact this has on markers of MASLD and MASH. In terms of linking key mechanistic pathways in the pathogenesis and progression of MASLD to MASH, mitochondrial function, oxidative and ER stress, gut microbiome composition and function, and inflammation have been considered where evidence permits. Nevertheless, this is not always the case, highlighting the need for further studies to establish causality and inform clinical practice. Additionally, the impact of metabolic phenotypes, encompassing insulin resistance, dyslipidemia, etc., are included since they reflect metabolic processes closely linked to the aforementioned mechanisms of MASLD progression [[118], [119], [120], [121], [122]]. A summary of the studies discussed in this section is provided in Table 1, wherein details of diet composition, the impact on liver-related parameters, relevant biochemical parameters, and outcomes related to the key mechanisms of MASLD progression are summarized.

**TABLE 1:** Characteristics of human dietary interventions included in the review.

Author, y	Duration and study type	Type and composition of diet (n)	Comparator diet and composition (n)	Liver-related parameters	Liver enzymes	Metabolic and other outcomes	Results confounded by weight loss
Low-carbohydrate diets							
Kirk, 2009	11-wk randomized clinical trial	Hypocaloric low-carbohydrate (n = 11): ≤ 60 g carbohydrates (∼10% EI), 75% fat, 15% protein	Hypocaloric high-carbohydrate (n = 11): ≥180 g carbohydrates (∼65% EI), 20% fat, 15% protein	Liver fat↓^1^ Fibrosis?	ALT ↔^1^ AST ↔^1^	Fasting glucose ↓^1^ Fasting insulin ↓^1^, greater in low-carbohydrate HOMA-IR ↓^1^, greater in low-carbohydrate	Yes
Haufe, 2011	6-mo randomized clinical trial	Hypocaloric low-carbohydrate (n = 84): ≤90 g carbohydrates, 30% fat, 0.8 g/kg protein	Hypocaloric low-fat (n = 86): ≤20% fat, 0.8 g/kg protein, remaining energy from carbohydrates	Liver fat ↓^1^ Fibrosis?	ALT ↓^1^ AST ↓^1^	Fasting glucose ↓^1^ Fasting insulin ↓^1^ HOMA-IR ↓^1^ Fasting TG ↓^1^ NEFA ↔^1^ TC ↔^1^ LDL-C ↔^1^ HDL-C ↔^1^	Yes
Kani, 2014	8-wk randomized clinical trial	Hypocaloric low-carbohydrate (n = 15): 45% carbohydrates, 35% fat, 20% protein	1) Hypocaloric diet (n = 15): 55% carbohydrates, 30% fat, 15% protein 2) Hypocaloric, low-carbohydrate, soy-containing diet (n = 15): Same as low-carbohydrate diet + 30 g soy nut daily	Liver fat? Fibrosis?	ALP ↓^1^ ALT ↓^1^ AST ↓^1^	Fasting insulin ↓, low-carbohydrate + soy Fasting TG ↓^1^ TC ↓^1^ LDL-C ↓^1^ CRP ↓, low-carbohydrate + soy group	Yes
Skytte, 2019	6-wk randomized crossover trial	Low-carbohydrate, high-protein (n = 28): 30% carbohydrates, 40% fat, 30% protein	Conventional diabetes diet (n = 28): 50% carbohydrates, 33% fat, 17% protein	Liver fat ↓ Fibrosis ?	?	HbA1c ↓, low-carbohydrate Fasting glucose ↓, low-carbohydrate AUC glucose ↓, low-carbohydrate AUC insulin ↓, low-carbohydrate Fasting TG ↓, low-carbohydrate TG AUC ↓, low-carbohydrate TC ↓, low-carbohydrate	No
Holmer, 2021	12-wk randomized controlled trial	Hypocaloric low-carbohydrate (n = 25): 10% carbohydrates, 50–80% fat, 15–40% protein	1) 5:2 diet (n = 25): 45–50% carbohydrates, 25% fat, 10–20% protein. On 2 nonconsecutive days, females consumed 500 kcal/d, males 600 kcal/d. On other 5 d, females consumed 2000 kcal/d, males 2400 kcal/d. 2) Standard of care (n = 24): Advised to reduce SFA, increase UFA, and eat 3 regular meals.	Liver fat ↓, is greater in low-carbohydrate and 5:2 diet Fibrosis ↓in 5:2 and standard of care	ALT ↓^1^ AST ↓, standard of care	HbA1c ↓, low-carbohydrate and 5:2 diet HOMA-IR ↓, low-carbohydrate, and 5:2 diet TC ↓, 5:2 diet LDL-C ↓, 5:2 diet HDL-C ↑, low-carbohydrate	Yes
London, 2024	4-d randomized crossover trial	Low-carbohydrate (n = 11): 14% carbohydrate, 70% fat, 16% protein	High-carbohydrate (n = 11): 70% carbohydrate, 14% fat, 16% protein	Liver fat ↓, low-carbohydrate Fibrosis?	?	Fasting glucose ↔ Hepatic insulin sensitivity ↑, low-carbohydrate Fasting TG ↓, low-carbohydrate TC ↓∗ LDL-C ↓, high-carbohydrate HDL-C ↔, low-carbohydrate but ↓ in high-carbohydrate	Yes
Ketogenic diet							
Tendler, 2007	6-mo clinical trial	Hypocaloric ketogenic (n = 5): <20 g carbohydrates daily, fat and protein not reported	n/a	Liver fat ↓ Fibrosis ↓ Necroinflammatory grade ↓	ALT ↔ AST ↔	Fasting glucose ↔ Fasting insulin ↔ HbA1c ↔ HOMA-IR ↔ TC ↔ Fasting TG ↔ LDL-C ↔ HDL-C ↔	Yes
Mardinoglu, 2018	14-d clinical trial	Isocaloric ketogenic (n = 10): 4% carbohydrates, 72% fat, 24% protein	n/a	Liver fat ↓ Fibrosis?	ALP ↓ AST ↓	Fasting insulin ↓ HOMA-IR ↓ Plasma VLDL ↓ Fasting TG ↓ IL-6 ↓ TNF-α ↓ FGF-21 ↓ Streptococcus, Lactococcus, Eggerthella species ↑ Ruminococcus, Eubacterium, Clostridium, Bifidobacterium ↓	Yes
Luukkonen, 2020	6-d clinical trial	Hypocaloric ketogenic (n = 10): ≤25 g (6%) carbohydrates, 64% fat, 28% protein	n/a	Liver fat ↓ Fibrosis ↔	ALP ↓ ALT ↔ AST ↔ GGT ↓	Fasting glucose ↓ Fasting insulin ↓ HOMA-IR ↓ Fasting TG ↓ TC ↔ LDL-C ↔ HDL-C ↔	Yes
Wolver, 2020 (abstract only)	6-mo clinical trial (interim analysis)	Not available (n = 30)	n/a	Liver fat ↓ Fibrosis ↓	ALT ↓ AST ↔	Not available	Yes
Rinaldi, 2023	8-wk clinical trial	Very low-calorie ketogenic diet (n = 33) 20–50 g carbohydrates, 15–30 g fat, 1–1.4 g/kg protein	n/a	Liver fat ↓ Fibrosis ↔	ALT ↓ GGT ↓	HOMA-IR ↓ Fasting glucose ↓ Fasting insulin ↓ Fasting TG ↓ TC ↓ LDL-C ↓ HDL-C ↑	Yes
Free sugars							
Volynets, 2013	6-mo pilot clinical trial	Reduced fructose diet (n = 10): 50% reduction in fructose intake	n/a	Liver fat ↓ Fibrosis?	ALT ↓ AST ↓ GGT ↓	Fasting glucose ↓ Fasting insulin ↓ HOMA-IR ↓ Fasting TG ↔ TC ↔ LDL-C ↔ HDL-C ↔ Intestinal permeability ↔ SIBO ↔ Bacterial endotoxin ↓	Yes
Jin, 2014	4-wk double-blind randomized controlled trial	3 x beverages with 33 g fructose daily (n = 9)	3x beverages with 33 g glucose daily (n = 12)	Liver fat ↔^1^ Fibrosis?	ALT ↔^1^ AST ↔^1^	Fasting glucose ↔^1^ Fasting insulin ↔^1^ HOMA-IR ↔^1^ Adipo-IR↓, glucose group; no change in fructose group Fasting triglycerides ↔^1^ Fasting NEFA ↓, glucose group; no change in fructose group CRP ↓, glucose group; no change in fructose group	No
Mager, 2015	6-mo nonrandomized controlled trial	Low-GI, low-GL, low fructose diet in children with MASLD (n = 12)	Low-GI, low-GL, low fructose diet in healthy children (n = 14)	Liver fat Fibrosis?	AST ↔^1^ GGT ↔^1^	Fasting glucose ↔^1^ HOMA-IR ↓, MASLD group Fasting TG ↓, MASLD group TC ↔^1^ LDL-C ↔^1^ HDL-C ↔^1^ CRP ↔^1^ TNF-α ↔^1^ IL-6 ↔^1^ IL-10 ↔^1^	No
Khodami, 2022	12-wk randomized controlled trial	Low-free sugar diet (n = 27): Free sugars <10% with no restriction on fat or protein, plus advice on a balanced diet (lean meat, low-fat dairy, whole grains, legumes, fruit, and vegetables)	Usual diet (n = 25): Balanced diet advice as per low-sugar group	Liver fat ↓, low-free sugar Fibrosis ↓, low-free sugar	ALT ↓^1^ AST ↔^1^ GGT ↔^1^	Fasting glucose ↓^1^ Fasting insulin ↓^1^ HOMA-IR ↓^1^ QUICKI ↑, low-free sugar Fasting TG ↓^1^ TC ↓^1^ LDL-C ↔^1^ HDL-C ↔^1^ TNF-α ↓, low-free sugar CRP ↓, low-free sugar	No^3^
Kord-Varkaneh, 2023	12-wk randomized controlled trial	16:8 + low-sugar diet (n = 27)): 8-h eating window daily with 16-h fasting, 55% carbohydrates with <3% free sugars, 30% fat, 15% protein; ≥9 servings of fruit and vegetables daily	Usual diet (n = 25) Usual diet but with same macronutrient composition as intervention group with no restriction on free sugar intake; ≥9 servings fruit and vegetables daily	Liver fat ↓, 16:8/low-free sugar Fibrosis?	ALT ↓, 16:8/low-free sugar AST ↓, 16:8/low-free sugar GGT ↓, 16:8/low-free sugar	Fasting glucose ↓, 16:8, low-free sugar Fasting insulin ↔^1^ HOMA-IR ↔^1^ Fasting TG ↓, 16:8, low-free sugar TC ↓, 16:8, low-free sugar LDL-C ↔^1^ HDL-C ↔^1^ CRP ↓, 16:8, low-free sugar	No^3^
Whole grains and fiber							
Malaguarnera, 2012	24-wk randomized controlled trial	Prebiotic supplementation (n = 34): *Bifidobacterium longum* plus fructo-oligosaccharides (Fos); diet and physical activity advice to achieve 1600 kcal/d, 55% carbohydrates, 20% fat, 25% protein	Placebo (n = 32): Placebo supplement plus same diet and lifestyle advice as prebiotic group	Liver fat ↓^1^, no difference between groups Fibrosis ↓^1^, no difference between groups Inflammation ↓^1^, no difference between groups MASH activity ↓, prebiotic + Fos	ALT ↓, prebiotic + Fos AST ↓, prebiotic + Fos	Fasting glucose ↓, prebiotic + Fos Fasting insulin ↓, prebiotic + Fos HOMA-IR ↓, prebiotic + Fos Fasting TG ↓, prebiotic + Fos TC ↓, prebiotic + Fos LDL-C ↓, prebiotic + Fos HDL-C ↑, prebiotic + Fos CRP ↓, prebiotic + Fos TNF-α ↓, prebiotic + Fos Serum endotoxin ↓, prebiotic + Fos	Yes
Bomhof, 2019	9-mo randomized controlled trial	Oligofructose prebiotic (n = 8): 8 g/d for 12-wk then 16 g/d for 24-wk	Maltodextrin placebo (n = 6)	Liver fat ↓, oligofructose Fibrosis ↔ MASH activity score ↓, oligofructose	ALT ↔^1^ AST ↔^1^ GGT ↔^1^	Fasting glucose ↓, oligofructose Fasting insulin ↔^1^ HOMA-IR ↔∗ Matsuda Index ↔^1^ Bifidobacterium ↑, oligofructose Clostridium cluster I + XI ↓, oligofructose	No
Dorosti, 2020	12-wk randomized controlled trial	Increased whole grain diet (n = 47) Instructed to obtain at least ½ cereal intake from whole grains plus eat 2–3 serves low-fat dairy, 5 serves fruit and vegetables, 2 serves lean protein	Usual grains (n = 47): Same diet advice as whole grain group but instructed to consume cereal intake from usual sources	Liver fat ↓, whole grain Fibrosis?	ALT ↓, wholegrains AST ↓, wholegrains GGT ↓, whole grains	Fasting glucose ↔^1^ Fasting insulin ↓, wholegrains HOMA-IR ↓, wholegrains Fasting TG ↔∗ TC ↓, wholegrains LDL-C ↓, wholegrains HDL-C ↑, wholegrains	No^1^3
Stachowska, 2022	2-mo clinical trial	High-fiber intervention (n = 27): Replacement of usual bread with 2 high-fiber rolls daily	n/a	Liver fat ↓ Fibrosis?	?	Serum propionate ↓ Serum acetate ↓ Serum butyrate ↔ Isobutyric acid ↓ Isovaleric acid ↓ 2-methylbutyric acid ↓	?
De Nucci, 2023	3-mo clinical trial	Increased vegetable intake (n = 24): Replaced 1 portion of carbohydrate-rich food with a portion of leafy green vegetables	n/a	Liver fat ↔ Fibrosis? FLI ↓ FAST ↓	ALT ↔ AST ↓ GGT ↓	Fasting glucose ↔ Fasting insulin ↔ HOMA-IR ↔ HbA1c ↓ Fasting TG ↓ TC ↔ LDL-C ↔ HDL-C ↔	Yes
***PUFA***							
Tanaka, 2008	12-mo clinical trial	2700 mg highly purified EPA daily (n = 23)	n/a	Liver fat ↓ Fibrosis ↓^2^ Inflammation ↓^2^ Ballooning ↓^2^ MASH activity score ↓^2^	ALT ↓ AST ↓ GGT ↔	Fasting glucose ↔ Fasting insulin ↔ HOMA-IR ↔ HbA1c ↔ Fasting TG ↔ TC ↓ NEFA ↓ HDL-C ↔	No
Sanyal, 2014	12-mo double-blind randomized controlled trial	1) Low-dose ethyl EPA (n = 82): 1800 mg/d 2) High-dose ethyl EPA (n = 86): 2700 mg/d	Placebo (n = 75)	Liver fat ↔ Fibrosis ↔ Inflammation ↔ Ballooning ↔ MASH activity score ↔	ALP ↔ ALT ↔ AST ↔	Fasting glucose ↔ Fasting insulin ↔ HOMA-IR ↔ HbA1c ↔ Fasting TG ↔ TC ↔ LDL-C ↔ HDL-C ↔ CRP ↔	No
Argo, 2015	12-mo double-blind randomized controlled trial	Combined EPA/DHA (n = 17): 3000 mg/d (EPA 35%, DHA 25%, 10% other n-3 fatty acids) plus advice to maintain a hypocaloric diet with <30% fat and to complete 150-min/wk aerobic exercise	Placebo (n = 17): Placebo (mostly soybean oil plus 8% fish oils) plus same diet and lifestyle advice as EPA/DHA group	Liver fat ↓ Fibrosis ↔ MASH activity score ↔	ALT ↔	Fasting glucose ↔ Fasting insulin ↔ HOMA-IR ↔ QUICKI ↔ Fasting TG ↔ TC ↔ LDL-C ↔	No^3^
Dasarathy, 2015	48-wk double-blind randomized controlled trial	Combined EPA/DHA (n = 18): 3600mg/d (2160 mg EPA + 1440 mg DHA) plus advice on healthy heart diet and to complete 30-min aerobic exercise x5 d/wk	Placebo (n = 19): Placebo (corn oil) plus same diet and lifestyle advice as EPA/DHA group	Liver fat ↔, reduced in placebo group Fibrosis ↔ Inflammation ↔, reduced in placebo group Ballooning ↔ MASH activity score ↔, reduced in placebo group	ALT ↔ AST ↔	Fasting glucose ↑ Fasting insulin ↔ HOMA-IR ↑ HbA1c ↑ Fasting TG ↔ TC ↔ HDL-C ↔	No
Li, 2015	6-mo randomized controlled trial	Combined EPA/DHA (n = 39): 50 mL supplement daily with 1:1 EPA/DHA plus advice on low-fat, low-cholesterol, or low-carbohydrate diet and to complete 30-min moderate exercise x 5 d/wk	Placebo (n = 39): Placebo plus same diet and lifestyle advice as EPA/DHA group	Liver fat ↓ Fibrosis ↓ Inflammation ↓ Ballooning ↓ Type IV procollagen ↓ Procollagen type III propeptide ↓	ALT ↓ AST ↓	Fasting glucose ↔ Fasting TG ↓ TC ↓ LDL-C ↔ HDL-C ↔ CRP ↓	Yes
Nogueira, 2016	6-mo randomized controlled trial	Combined AHA/EPA/DHA (n = 27): 945 mg/d (64% ALA, 21% EPA, 16% DHA)	Placebo (n = 28): Mineral oil capsule daily	Liver fat ↔ Fibrosis ↔ Inflammation↔ Hepatocyte ballooning ↔	ALT ↔ AST ↔ GGT ↔	Fasting glucose ↔ Fasting insulin ↔ HbA1c ↔ TC ↔ LDL-C ↔ HDL-C ↔ Fasting TG ↓	No
Okada, 2018	6-mo randomized controlled trial; extension of Nogueira et al.	Combined AHA/EPA/DHA (n = 27): 945 mg/d (64% ALA, 21% EPA, 16% DHA)	Placebo (n = 28): Mineral oil capsule daily. Note: These patients were not included in the proteomic and lipidomic analysis	As above	As above	As above Improved hepatic proteome Improved systemic lipidomic markers of endoplasmic reticulum stress, lipogenesis, and mitochondrial function	No
***MUFA***							
Bozzetto, 2012	8-wk randomized controlled trial	1) High-MUFA diet (n = 8): 40% carbohydrates, 10 g fiber/1000 kcal, GI 95%, 42% fat, 28% MUFA, 7% SFA, 4% PUFA, 18% protein 2) High-MUFA diet plus exercise (n = 9): Diet as above plus 2 x 45-min exercise sessions weekly at 70% baseline VO_2_ max.	1) High-carbohydrate, high-fiber, low-GI diet (n = 9): 52% carbohydrates, 28g fiber/1000kcal, G.I. 60%, 30% fat, 16% MUFA, 7% SFA, 4% PUFA, 18% protein 2) High-carbohydrate, high-fiber, low-GI diet plus exercise (n = 10): Diet as above plus exercise sessions as per high-MUFA plus exercise group.	Liver fat ↓, high-MUFA, and high-MUFA + exercise Fibrosis?	ALT ↔ AST ↓, high-MUFA, and high-MUFA + exercise	Fasting glucose ↔ Fasting insulin ↔ HOMA-IR ↔ HbA1c ↓, high-MUFA diet Fasting TG ↔ TC ↔ LDL-C ↔ HDL-C ↔	No
Bjermo, 2012	10-wk randomized controlled trial	Isocaloric high-PUFA diet (n = 30): Advised to change quality of dietary fat intake without altering quantity or type/amount of carbohydrate or protein. Consumed ∼15% linoleic acid.	Isocaloric high-SFA diet (n = 29): Advised to change quality of dietary fat intake without altering quantity or type/amount of carbohydrate or protein	Liver fat ↓ Fibrosis?	ALT ↔ GGT ↔	Fasting glucose ↔ Fasting insulin ↔, increased in SFA HOMA-IR ↔ AUC glucose ↔ AUC insulin ↔ FGF-21 ↔ CRP ↔ IL-1β ↔ IL-6 ↔ IL-10 ↔	No
Ryan, 2013	6-wk randomized crossover trial	Mediterranean diet (n = 12): 40% carbohydrates, 40% fat, 20% protein	Low-fat, high-carbohydrate diet (n = 12): 50% carbohydrates, 30% fat, 20% protein	Liver fat ↓ Fibrosis?	ALT ↔ GGT ↔	Fasting glucose ↔ Fasting insulin ↓ HOMA-IR ↓ Fasting TG ↔ HDL-C ↔	No
Errazuriz, 2017	12-wk randomized controlled trial	1) Isocaloric high-MUFA diet (n = 15): 28% EI from MUFA, with 50% of this from olive oil 2) Isocaloric high-fiber diet (n = 15): 20 g fiber per 1000 kcal, with minimum of 1 cup cooked beans/d, and max. 72g fiber	Usual diet (n = 13)	Liver fat ↓, high-MUFA Fibrosis?	?	OGTT-derived glucose ↔ OGTT-derived insulin ↔ OGTT-derived C-peptide ↔ Hepatic insulin sensitivity ↑, high-MUFA (within-group) Total insulin sensitivity ↑, high-MUFA (within-group)	No
Luukkonen, 2018	3-wk randomized clinical trial	Hypercaloric diets with +1000 kcal from varying nutrients: 1) High-SFA (n = 14): 76% SFA, 57% MUFA, 22% PUFA from coconut oil, butter, and blue cheese 2) High-UFA (n = 12): 21% SFA, 57% MUFA, 22% PUFA from olive oil, pesto, pecan nuts, and butter 3) High simple sugars (n=12): 100% simple sugars from fruit juice, sugar-sweetened beverages, and candy	n/a	Liver fat ↑^1^, greatest increase in SFA Fibrosis?	ALT ↑, SFA AST ↑, SFA	Fasting insulin ↑, SFA HOMA-IR ↑, SFA NEFA ↓ during hyperinsulinemia, UFA, and simple sugars Fasting TG ↔∗ LDL-C ↑, SFA HDL-C ↑, SFA De novo lipogenesis ↑, simple sugars Serum ceramides ↑, SFA	No
George, 2022	12-wk randomized controlled trial	Mediterranean diet (n = 19): 33% carbohydrates, 44% fat, 20% protein, <5% alcohol	Low-fat diet (n = 23): 50% carbohydrates, 30% fat, 20% protein	Liver fat ↓, low-fat Fibrosis ↔^1^	ALT ↓, low-fat AST ↓, low-fat GGT ↓, low-fat	Fasting glucose ↔^1^ Fasting insulin ↔^1^ HOMA-IR ↔^1^ Fasting TG ↔^1^ TC ↔^1^ LDL-C ↔^1^ HDL-C ↔^1^	Yes, low-fat group
Montserrat-Mesquida, 2022	6-mo clinical trial	High-Mediterranean diet adherence (n = 36): Selected from 3 intervention groups, see note^4^	Low-Mediterranean diet adherence (n = 31)	Liver fat ↓ Fibrosis?	ALT ↓ AST ↓ GGT ↓∗	Fasting glucose ↓ HbA1c ↔ Fasting triglycerides ↔ TC ↔ LDL-C ↔ HDL-C ↔ CRP ↔ IL-1β ↓ IL-6 ↓ TNF-α ↔ CK-18 ↓ GSH ↓ CAT ↔ SOD ↑ PBMC TLR4 mRNA expression ↓	Yes
Quetglas-Llabrés, 2023	12-mo randomized controlled trial	High-Mediterranean diet adherence (n = 35): Selected from 3 intervention groups, see note^4^	Low-Mediterranean diet adherence (n = 32)	Liver fat ↔ Fibrosis?	ALT ↓ AST ↓ GGT ↔	Fasting glucose ↔ HbA1c ↔ Fasting TG ↓ TC ↓ LDL-C ↔ HDL-C ↔ CRP ↓ IL-1β ↔ IL-6 ↔ TNF-α ↔ CK-18 ↓ CAT ↓ MDA ↓^1^ SOD ↔ Zonulin ↓^1^ Endotoxin ↓^1^	Yes
Quetglas-Llab rés, 2024	24-mo randomized controlled trial	High-Mediterranean diet adherence (n = 20)	Low-Mediterranean diet adherence (n = 20)	Liver fat ↓, high adherence Fibrosis?	ALT ↓ AST ↓ GGT ↓ All in high adherence only	Fasting glucose ↓ HbA1c ↔ Fasting TG ↓^1^ TC ↓^1^ LDL-C ↓^1^ HDL-C ↑^1^ CRP ↔ CK-18 ↓^1^ GPx ↓^1^ GRd ↓ GSH ↓ CAT ↓ SOD ↓ PBMC TLR4 mRNA expression ↓ ROS production by PBMCs stimulated with zymosan and LPS Plasma MDA ↓∗ Erythrocyte MDA ↓ oxLDL ↓	Yes

↑ significant increase; ↓ significant decrease; ↔ no significant change;? not assessed or reported.

Abbreviations: ALT, alanine transaminase; ALP, alkaline phosphatase; AST, aspartate transferase; CAT, catalase EI, energy intake; FAST, FibroScan AST score; FGF-21, fibroblast growth factor 21; FLI, fatty liver index; GGT, gamma-glutamyl transferase; GI, glycemic index; GPx, glutathione peroxidase; GRd, glutathione reductase; GSH, total glutathione; LDL-C, low-density lipoprotein cholesterol; MASLD, metabolic dysfunction-associated steatotic liver disease; MASH, metabolic-dysfunction associated steatohepatitis; MDA, malondialdehyde; NEFA, nonesterified fatty acids; SIBO, small intestinal bacterial overgrowth; SOD, superoxide dismutase; TC, total cholesterol; TNF-α, tumor necrosis factor alpha

1 Results observed in all groups

2 In 7/23 patients who consented to liver biopsy

3 Adjusted results which controlled for confounding by weight loss

4 Initially compared 3 different intervention groups (Mediterranean diet + high-meal frequency, Mediterranean diet + physical activity, and conventional diet). Changed analysis to high- vs. low-Mediterranean diet adherence when no differences were observed between groups, with diet type, meal frequency, and physical activity as co-variables

### Modulation of dietary carbohydrate intake

#### Low-carbohydrate diets

Low-carbohydrate diets (LCDs), in particular the ketogenic diet (KD), are hypothesized to mitigate MASLD progression by targeting key metabolic pathways. This dietary approach is attractive as limiting carbohydrate consumption reduces blood glucose, thereby lowering insulin secretion and DNL [123]. By reducing DNL, LCDs may alleviate ER stress, a key driver of the MASLD-MASH transition, as excessive DNL has been shown to overload the ER, impair protein folding, and trigger the unfolded protein response. Additionally, limiting dietary fructose within the context of an LCDs may help to maintain gut barrier function and alter gut microbial composition, potentially lowering systemic inflammation via decreased endotoxin translocation from the gut – a critical contributor to MASLD progression via gut-liver axis dysfunction. However, although these mechanisms are consistent with metabolic improvements observed in some LCD intervention studies, direct evidence linking dietary carbohydrate restriction to the mechanisms driving MASH remains limited.

Evidence of the effects of a low-carbohydrate, nonketogenic diet, where carbohydrate intake is limited to ∼50–140g daily, is heterogeneous due to variations in diet composition and study duration. LCDs, by this definition, have been shown to reduce liver fat [[123], [124], [125], [126], [127]], whereas the impact on MASH-related inflammation and fibrosis and the mechanisms driving these outcomes is understudied. Compared with the standard of care and the 5:2 diet, a form of intermittent fasting, a hypocaloric LCD where carbohydrate intake was limited to 10% of energy intake did not reduce liver fibrosis as assessed by liver elastography [128]. This lack of effect was observed even with significant weight loss.

On the other hand, LCDs have been shown to improve markers of glucose and insulin homeostasis, such as HbA1c and HOMA-IR, as well as blood lipid profiles [[123], [124], [125], [126]]. However, as most studies involve calorie restriction and/or significant weight loss, these effects cannot be ascribed to carbohydrate restriction alone. Indeed, compared with a low-fat or standard hypocaloric diet, LCDs generally do not offer superior benefits for liver-related parameters or for metabolic health. For example, liver fat content, fasting insulin and glucose, HOMA-IR, and fasting triglycerides were reduced to a similar extent following 6-mo of a reduced carbohydrate, hypocaloric diet (1600 kcals, 100–110g carbohydrates, 70–80g fat daily) compared with a reduced fat, hypocaloric diet (1700 kcals, 200–230g carbohydrates, 40–60g fat daily) [124]. A greater reduction in LDL-cholesterol was observed in the reduced fat group, albeit with a greater reduction in saturated fat intake, which is a major determinant of LDL-cholesterol concentrations. Of note, there was a 2-fold greater reduction in TGF-β1 in the reduced carbohydrate group, which plays a role in the pathogenesis of fibrosis, suggesting a potential advantage of carbohydrate restriction in fibrogenesis. Nonetheless, further work is required to determine whether LCDs without weight loss could be used to attenuate the progression of MASLD to MASH. The results of an ongoing randomized controlled trial investigating the impact of a moderately restricted carbohydrate diet on liver fat and fibrosis in normal-weight individuals may provide some insights [129].

Some studies suggest that the KD could attenuate the progression of MASLD to MASH by reducing inflammation and improving mitochondrial function. β-hydroxybutyrate, the main ketone body produced during the ketogenic diet, has been linked with inhibition of the NLRP3 inflammasome, which shows higher expression in MASH compared with MASLD [111,130]. Additionally, animal and cell models suggest that β-hydroxybutyrate could improve mitochondrial biogenesis and function by inducing peroxisome proliferator-activated receptor gamma coactivator 1-alpha in the liver [131], potentially mitigating ROS production and organelle dysfunction in hepatocytes. However, some studies have observed increased hepatic insulin resistance in mice with KD feeding [131,132]. In humans, a short-term KD (6-d) of ≤25 g/d was shown to favorably alter hepatic mitochondrial fluxes to promote ketogenesis rather than synthesis of TAG [133]. These effects may indirectly alleviate ER stress by reducing lipid overload. In this study, hepatic lipid content was reduced by 31%. Liver stiffness remained unchanged, and the AST/ALT ratio increased, suggesting that hepatocellular injury may have been caused by the rapid weight loss of 3%.

The severity of MASLD and progression to MASH is associated with higher levels of IL-6 and TNF-α [134,135], and plasma cytokine concentrations were reduced after 14 d of a KD consisting of 23 to 30 g carbohydrates/d [136]. This may be linked to a number of factors, such as reduced inflammasome activation, ER stress and lipotoxicity, and improved mitochondrial function, although these mechanistic insights are lacking. Despite minimal weight loss in this study (1.8%), hepatic lipid content was also reduced by 44%, and HOMA-IR was significantly reduced. Unfortunately, the study lacked a control group, and diets were not matched to baseline in terms of macronutrient quality; therefore, overall improvements in diet quality may have influenced the results. Additionally, although liver biopsies were available for some of the cohort, effects of the intervention on markers of MASH activity were not assessed, making it difficult to infer benefits for preventing disease progression. Tendler et al. [137] performed liver biopsies after 6-mo of a KD of <20 g carbohydrates/d. They observed a significant reduction in hepatic necroinflammatory grade and an appreciable but non-significant reduction in fibrosis score. However, the results were confounded by weight loss, and the study did not have a comparator arm. Using a very low calorie KD (VLCKD), Wolver et al. [138] achieved a significant reduction in liver fibrosis after 6-mo, although these results were presented as a conference abstract; therefore, the detailed methods and final results were not available. A similar study reported that liver fibrosis was reduced in males only [139].

Changes to the gut microbiota composition and derivatives thereof may be relevant to the progression of MASLD. Indeed, we speculate that the downstream effects of gut-derived metabolites are probably most important, e.g., short-chain fatty acids (SCFA), secondary bile acids, etc. Other reviews have addressed this complex area [80], providing insights beyond that within the dietary studies captured in Table 1. Nevertheless, within this context, in the previously mentioned 14-d KD trial, an increase in serum folate was observed, which was related to greater abundance of the folate-producing species Streptococcus and Lactococcus rather than changes to dietary intake [136]. Given the inverse association between serum folate and MASH severity, this finding highlights a potential link between KD-driven microbiota changes and MASLD progression [140]. In mice, dietary folic acid intake attenuated inflammation and fibrosis in MASH [142], and macrophage expression of IL-6 and TNF-α was increased with folate deficiency [141]. However, the mechanisms remain speculative, as reduced dietary carbohydrate intake may have enhanced intestinal folate absorption, and as highlighted, the study did not have a control group.

Further supporting a role for KD-associated microbiota changes, a recent study demonstrated that fecal microbiota transplantation (FMT) from individuals consuming either a balanced diet or KD for 8-weeks in germ-free mice revealed a small intestine-specific effect of KD microbiota in reducing Th17 cell accumulation [143]. These observations were linked to depletion of Bifidobacterium. Th17 cells are implicated in intestinal inflammation and metabolic endotoxemia, recognized contributors to MASLD progression. By supplementing HFD-fed mice with ketone esters, they demonstrated that ketone bodies are central to the shifts in Bifidobacterium abundance and the subsequent reduction in Th17 cells. Although this study did not include patients with MASLD, the observed immunological shifts highlight a potential mechanism by which the KD may modulate gut-liver axis inflammation to delay disease progression. Further work is required to confirm these findings in humans and to fully understand the impact of the KD on the microbiome and their metabolites in MASLD, both in terms of composition and function, since both factors are relevant to the progression to MASH.

#### Free sugars

Whereas LCD and KD approaches broadly target carbohydrate restriction, the role of free sugars, particularly fructose, warrants closer examination given its specific mechanistic links to MASLD progression. Free sugars refer to monosaccharides and disaccharides which are added to foods and drinks by the consumer or manufacturer, or those naturally present in foods and drinks such as honey and fruit juice. Fructose, which is largely consumed as high-fructose corn syrup and as sucrose, is particularly harmful to the liver and has been implicated in a number of processes involved in the MASLD-MASH transition. Indeed, fructose promotes DNL, ER stress, and alters gut microbiota composition and function. Observational studies provide strong evidence of these links, with daily fructose consumption being associated with increased hepatic fibrosis and increased MASH histological activity in older patients (>48 y) [144,145].

Despite convincing evidence outlined previously, few dietary interventions specifically focused on reducing fructose consumption in MASLD have been conducted, and even fewer have focused on outcomes related to MASH. In the context of metabolic health and inflammation, a 6-mo intervention of low fructose and low-glycemic index/load diet in children and adolescents with MASLD did not improve circulating IL-6 or TNF-α; however, HOMA-IR and plasma triglycerides were reduced [146]. On the other hand, plasma CRP and cardiometabolic profiles were improved in adolescents when fructose-containing drinks were replaced with glucose drinks for 4-wk [147]. Similar results were reported in adults, wherein 6-mo of a 50% reduction in dietary fructose consumption significantly reduced AST, ALT, fasting glucose and insulin, and HOMA-IR. This study also assessed the impact of reduced fructose consumption on intestinal permeability, a key mechanism linking dietary fructose to hepatic inflammation [148]. Although the intervention did not affect intestinal permeability and did not reduce intestinal bacteria overgrowth, plasma endotoxin levels decreased, highlighting the potential benefit of reduced fructose consumption for reducing systemic inflammation.

More broadly, low-sugar diets have also been investigated in MASLD. In a 12-wk trial, a low-free sugar diet significantly reduced hepatic fibrosis, CRP, and TNF-α in MASLD [149]. This reduction remained significant when adjusted for BMI and other confounding factors. A significant reduction in carbohydrate intake was observed in the intervention group on account of the sugar restriction; however, both groups maintained consumption of healthy carbohydrates and a high-fiber intake, suggesting that the change in sugar intake mediated most of the benefits. Nonetheless, it is plausible that the maintenance of a high fiber intake acted synergistically with the sugar restriction, further contributing to the observed reduction in hepatic fibrosis and inflammatory cytokines by targeting the key mechanisms involved in MASLD progression, i.e., altered gut barrier integrity and microbiome composition, ER stress and downstream lipotoxicity, and inflammatory signaling. Similarly, a low-sugar diet combined with time-restricted eating (16:8) led to significant reductions in fibrosis, liver enzymes, plasma triglycerides, cholesterol, and CRP after 3-mo [150]. The intervention group experienced an average weight loss of 3.8%, and energy intake was reduced in both groups; however, these results remained significant when adjusted for BMI and energy intake. Future work focused on defining the precise mechanisms underlying the benefits of low-sugar diets is warranted.

#### Wholegrains and fiber

Whereas the removal of simple sugars from the diet may attenuate the progression of MASLD, the addition of high-fiber, no-digestible carbohydrates, such as whole grains and vegetables, could also be beneficial. In a study investigating the impact of switching one carbohydrate-dense food for a portion of green leafy vegetables, fatty liver index (used to predict fatty liver) and the FibroScan-AST (FAST) score (measure of the risk of progressing to MASH) both improved [151]. Metabolic markers such as HbA1c, triglycerides, and AST were also improved. Decreases in BMI and fat mass were reported, which, although beneficial to those involved in the study, may have caused the decreases in FAST, FLI, and metabolic markers rather than the specific effects of increasing green leafy vegetable consumption. However, it does highlight that simple changes can have beneficial effects in slowing progression to MASH. This study did not investigate specific mechanisms of progression from MASLD to MASH. However, consumption of green leafy vegetables has been shown elsewhere to protect mitochondrial function by reducing free radicals which cause oxidative stress, due to the antioxidants in green leafy vegetables such as vitamin C, bioflavonoids, xanthophylls, carotenes, and anthocyanidins [152]. Therefore, it could be speculated that these components, through protection of mitochondrial function, may slow progression from MASLD to MASH.

A study that substituted regular bread for high-fiber buns for 2-mo demonstrated that this simple switch decreased liver fat in individuals with MASLD as assessed by FibroScan [153]. However, this reduction in liver fat was only noted in 9 of 27 participants. A decrease in BMI was also reported despite no significant reductions in calorie intake. Changes in the serum metabolome, including decreases in acetate, 2-methylbutyric acid, isovaleric, and propionate, as well as increases in choline and proline, indicate that these effects could be mediated by changes in the serum microbiota metabolome influencing the condition of the liver. A similar larger study involving 112 people with MASLD aimed to study the benefits of whole grains in the diet [154]. The intervention group was instructed to obtain at least half of their cereal intake from whole grain sources, whereas the control group was instructed to consume their usual cereals for 12-wk. The intervention group had reduced hepatic steatosis as assessed by liver ultrasound and reduced liver enzymes concentrations (ALT, ALP, gamma-glutamyl transferase (GGT)) in comparison to control. Although these studies highlight the beneficial impacts of increasing fiber in terms of reducing steatosis, they failed to demonstrate the ability of these changes in diet to decrease liver stiffness, inflammation, intestinal permeability, and gut microbiome and immune cell function, important components in the progression of MASLD to MASH.

High-fiber intake may positively influence MASLD by altering the gut microbiome composition, thereby reducing the development of inflammation and liver injury. Within this context, the administration of prebiotics in the form of indigestible dietary fibers has been explored. Examples of these include polysaccharides such as inulin and oligosaccharides such as lactulose. Prebiotics function to selectively promote the proliferation of the gut microbiota [155]. A pilot clinical trial investigated the use of oligofructose in patients with MASH. One of their primary findings was an overall decrease in NAFLD activity score (NAS score), which is a combined grading system of steatosis, lobular inflammation, and hepatocellular ballooning [156]. Liver steatosis and lobular inflammation also improved; however, the latter did not reach statistical significance. Increases in Bifidobacterium and decreases in Clostridium cluster XI bacteria were also noted.

A similar study that administered *Bifidobacterium longum* with fructo-oligosaccharides to patients with MASH for 24 wk measured decreases in AST, LDL-cholesterol, TNF-α, HOMA-IR, serum endotoxin, steatosis, fibrosis, and NAS score, indicating a decrease in inflammation and fibrosis and potentially a delay in the worsening of MASH compared with control [157]. Increases in Bifidobacterium have been shown to improve intestinal barrier integrity by stabilizing claudins at tight junctions in humans and mouse models, therefore potentially slowing the progression from MASLD to MASH [158].

### Modulation of dietary fat intake

As highlighted earlier, preclinical work demonstrates that different fatty acids exert distinct biological effects that can either exacerbate or mitigate MASLD progression. This emphasizes that the quality of dietary fat is as critical as the quantity. Strategies for altering dietary fat intake can, therefore, involve the addition, removal, and/or substitution of different fat types. As such, this section comprises an integrated discussion on modulating dietary fat intake for the management of MASLD.

Numerous n-3 PUFA supplementation studies have been conducted in MASLD. Mechanistically, 6-mo supplementation with 945 mg combined alpha-linoleic acid, EPA, and DHA was shown to modulate hepatic proteomic and plasma lipidomic markers of lipogenesis, ER stress, and mitochondrial function in patients with MASH, suggesting a benefit for preventing disease progression [159]. However, it is unclear whether this translates into a meaningful improvement in liver histology, as clinical trials have reported conflicting results, largely due to variations in dosing strategies and study duration (as summarized in Table 1). In a trial comparing EPA plus DHA compared with placebo in MASH, liver histology was significantly improved compared with controls after 6-mo [160]. ALT, AST, plasma triglycerides, total cholesterol, and CRP were reduced in the intervention group, as well as the fibrotic biomarkers type IV collagen and procollagen type III propeptide. However, the exact dose of EPA/DHA used was not stated, and reductions in BMI and concurrent lifestyle advice confounded the results. Another study reported that 2700 mg highly purified EPA for 12-mo improved histological features of MASH; however, only 7 of 23 participants consented to liver biopsy, and the study did not have a control group [161]. Further trials of 6- to 12-mo with doses ranging from ∼1000 to 3600mg EPA/DHA failed to induce significant changes in liver histology [[162], [163], [164], [165]]. These results suggest that the simple addition of fatty acids alone is likely not sufficient for the management of MASLD or MASH. Whether this is due to lack of effect on the mechanisms driving disease progression is unclear from the available evidence.

The impact of reducing or altering dietary SFA intake is intricately linked to what replaces these fatty acids in the diet. This concept was nicely illustrated by Bjermo et al., who demonstrated that 10-wk of an isocaloric diet high in n-6 PUFA compared with SFA, without other changes to macronutrient intake, significantly reduced liver fat and improved metabolic status as determined by fasting insulin, total/HDL-cholesterol ratio, LDL-cholesterol, and triglycerides [166]. Further, the n-6 PUFA diet did not affect a range of inflammatory and oxidative stress markers, including plasma CRP, IL-1β, IL-6, IL-10, IL-1 receptor antagonist, and TNF receptor 2, or urinary 8-iso-prostaglandin 2α and 15-keto-13,14-dihydro-prostaglandin 2α. Mechanistically, this reflects the ability of n-6 PUFA to mitigate ER stress and reduce hepatic lipotoxicity by decreasing lipolysis and ceramide accumulation, processes exacerbated by SFA [167]. Moreover, PUFA overfeeding studies confirm that, unlike SFA and simple sugars, PUFAs do not promote DNL or insulin resistance to the same extent [167]. However, most participants in this study had <5% liver fat and therefore did not meet the criteria for MASLD. Nonetheless, the effects observed may be more pronounced in those with higher liver fat.

Positive outcomes have also been reported with isocaloric high-MUFA (HMUFA) interventions. However, as with PUFA, human clinical data isolating the impact of MUFA on mechanistic pathways in MASH is limited. Bozzetto et al. showed that a HMUFA diet (28% MUFA), with or without exercise, significantly reduced liver fat compared with a high-carbohydrate/high-fiber/low-glycemic index (GI) diet after 10-wk [166]. Both diets also involved a reduction of saturated fat intake from 13% to 7% of total energy intake. In terms of metabolic outcomes, HbA1c was significantly reduced in the MUFA group, but fasting glucose and lipid profiles were unchanged in all groups, as were liver enzymes. Similarly, a weight-maintaining HMUFA intervention (28% MUFA) for 12-wk in individuals with prediabetes reduced liver fat by 17% compared with a high-fiber and habitual/control diet [168]. Within-group improvements were reported for hepatic and total insulin sensitivity in the MUFA group only.

The Mediterranean diet (MD), characterized by HMUFA intake, the replacement of saturated fat with unsaturated fat, and consumption of high-fiber, complex carbohydrates, incorporates multiple dietary principles that have independently demonstrated benefits in MASLD. Although the MD is generally recommended for the treatment of MASLD, evidence directly linking the diet to the key mechanisms in humans underlying the progression to MASH remains limited. Studies have shown that the MD reduces plasma markers of oxidative stress and inflammation in MASLD [[169], [170], [171]]. ROS production by PBMCs activated with LPS and zymosan was reduced in individuals with greater MD adherence [170]. TNF-α and IL-6 were also reduced, although only IL-6 reached significance [171]. These effects were consistent with the observed reduction in TLR4 expression [170,171], providing some mechanistic evidence for these effects. However, further confirmation of these findings is required in larger populations.

Regarding gut function, a 16-wk MD intervention in MASLD showed no significant changes in intestinal permeability [172]. Conversely, a 12-wk MD intervention reduced plasma zonulin and lipopolysaccharide binding protein (LBP), effects partly mediated by SCFA, albeit with significant interindividual variation [173]. Interestingly, baseline SCFA and permeability markers predicted intervention success, highlighting that personalized approaches might improve dietary outcomes. However, this study did not include individuals with MASLD and included females only.

Liver-specific and metabolic outcomes are also inconsistent. In a 6-wk crossover trial, the MD significantly reduced liver fat and improved insulin sensitivity compared with a low-fat, high-carbohydrate diet, even without weight loss [174]. Conversely, in the 12-wk Mediterranean Dietary Intervention for Patients with Non-Alcoholic Fatty Liver Disease (MEDINA) trial, only the low-fat diet led to a reduction in liver fat and HOMA-IR, whereas no change was seen in the MD group [175]. However, the low-fat group experienced a mean weight lossof 4.5% whereas weight increased slightly in the MD group. Liver stiffness remained unchanged in both groups. Collectively, these results suggest that whereas the MD is a holistic dietary approach combining multiple dietary strategies for MASLD treatment, the specific effects on MASH-related mechanisms are not fully understood. Further work will help to elucidate these effects and establish whether the MD can prevent or delay disease progression.

### Summary

Significant weight loss remains the cornerstone of dietary therapy in MASLD, and various dietary strategies are successful in this regard once this weight loss is achieved and maintained. It is, therefore, difficult to determine whether the benefits are due to the specific removal of SFA and/or fructose per se or a result of the accompanying weight loss, as seen with LCD and the KD. Independent of body weight changes, encouraging results for MASLD and MASH have been reported with some dietary strategies, including low-sugar and high-fiber diets, as well as prebiotics (see Table 1). In light of the evidence surrounding the role of SFA and fructose in driving MASH-related inflammation and fibrosis, reducing their intake is a priority. Further work is also required to determine the independent and additive impact of reducing consumption of these dietary triggers on disease progression, as at present, few trials have investigated this. In terms of replacing these dietary triggers, replacement of SFA with MUFA or PUFA has demonstrated benefits for hepatic steatosis, insulin sensitivity, and lipid profiles. However, these findings are often limited to short-term interventions and outcomes that do not address all of the mechanisms underpinning MASLD progression. The MD appears promising for reducing oxidative stress, inflammation, and liver fat; however, mechanistic evidence linking these effects to the progression of MASH remains scarce. Importantly, findings are often confounded by interindividual variation and small sample sizes. Collectively, although dietary strategies hold promise in delaying disease progression, further well-designed studies are needed to establish their mechanistic impacts and long-term efficacy.

## Future Directions

Considerable preclinical evidence implicates SFA and fructose in driving multiple mechanisms linked to the MASLD-MASH-fibrosis transition, and further work will undoubtedly elucidate the details of these mechanisms further, particularly with the greater application of big data techniques and artificial intelligence in the study of the microbiome and EV’s, as has been done for cancer [176,177]. However, data are lacking with regards to the combined effects of SFA and fructose in these same mechanisms. Therefore, the question of whether these effects are additive or synergistic remains unanswered. Furthermore, translation of this data into humans is lacking in terms of whether specifically reducing the intake of these nutrients can modulate these pathological processes to prevent disease progression.

Dietary interventions in MASLD to date have also largely failed to consider the background genotype and phenotype of the participants, which are significant modulators of the response to dietary intervention and partly form the basis of precision nutrition interventions [[178], [179], [180], [181]]. With respect to genotype, importantly, the patatin-like phospholipase domain containing 3 (PNPLA3) I148M variant, which has a global prevalence of 30 to 50%, is known to impair hepatic lipid metabolism and mitochondrial function, contributing to the development of steatosis and liver injury [182,183]. It is probable that the impact of both feeding SFA and fructose as metabolic ‘hits’ or insults is much greater in individuals carrying PNPLA3 variants. Under ketogenic conditions, it has been shown that levels of this variant protein are reduced [133,182]. Through a number of steps, including lipolysis of excess triglycerides, increased β-oxidation, and mitochondrial redox state, this perpetuates mitochondrial dysfunction and, eventually, liver injury [182]. Similarly, the C47T polymorphism of the *SOD2* gene is associated with more advanced fibrosis in MASLD, likely due to reduced oxidative stress protection, further demonstrating the relevance of genotype to the progression of MASLD [184]. Thus, genotypic variation may act to predispose the liver to be more adversely affected by the multiple hits involved in MASLD progression. Although this is an emerging area that needs to be further explored, this study highlights that the nature of the most appropriate dietary intervention may vary depending on genetic background and, therefore, should be considered in the future.

While not in subjects with MASLD, the impact of phenotype on the response to dietary intervention, according to insulin-resistant phenotypes, was elegantly demonstrated by Trouwborst et al. in the PERSON study [185]. In this study, either muscle- or liver- insulin-resistant phenotypes were determined prior to randomization. Participants were then assigned to either a low-fat, high-protein, high-fiber (LFHP), or HMUFA diet intervention for 12-wk. Those with muscle-insulin resistance showed greater improvements in metabolic health on the LFHP diet, whereas those with liver-insulin resistance had a greater response to the HMUFA diet. This emphasizes that superior cardiometabolic health improvements may be obtained using precision nutrition approaches that incorporate greater characterization of background participant phenotypes. Similar investigations in MASLD would help to elucidate exactly how nutrient intake can be modulated to prevent disease progression and could help to determine the optimal dietary strategy for each stage/phenotype of the disease.

## Author contributions

The authors’ responsibilities were as follows – SMM, AJK, MBNíC wrote the paper; SMM, AJK contributed equally to the writing of the paper; CES, SN, HMR read, revised, and approved the paper; and all authors: have read and approved the final manuscript.

## Funding

This work was supported by Science Foundation Ireland Frontiers Award Programme (SFI 19/FFP/6625) and the Higher Education Authority of Ireland COVID-19 Research Extension Response Award.

## Conflict of interest

The authors report no conflicts of interest.
